# Case Report: Phenotype-Driven Diagnosis of Atypical Dravet-Like Syndrome Caused by a Novel Splicing Variant in the *SCN2A* Gene

**DOI:** 10.3389/fgene.2022.888481

**Published:** 2022-05-31

**Authors:** Artem Sharkov, Peter Sparber, Anna Stepanova, Denis Pyankov, Sergei Korostelev, Mikhail Skoblov

**Affiliations:** ^1^ Genomed Ltd., Moscow, Russia; ^2^ Veltischev Research and Clinical Institute for Pediatrics of the Pirogov Russian National Research Medical University, Moscow, Russia; ^3^ Research Centre for Medical Genetics, Moscow, Russia

**Keywords:** SCN2A, functional analysis, deep phenotyping, Dravet syndrome, Kabuki syndrome

## Abstract

Febrile-associated epileptic encephalopathy is a large genetically heterogeneous group that is associated with pathogenic variants in *SCN1A*, *PCDH19*, *SCN2A*, *SCN8A*, and other genes. The disease onset ranges from neonatal or early-onset epileptic encephalopathy to late-onset epilepsy after 18 months. Some etiology-specific epileptic encephalopathies have target therapy which can serve as a clue for the correct genetic diagnosis. We present genetic, clinical, electroencephalographic, and behavioral features of a 4-year-old girl with epileptic encephalopathy related to a *de novo* intronic variant in the *SCN2A* gene. Initial NGS analysis revealed a frameshift variant in the *KDM6A* gene and a previously reported missense variant in *SCN1A*. Due to lack of typical clinical signs of Kabuki syndrome, we performed X-chromosome inactivation that revealed nearly complete skewed inactivation. Segregation analysis showed that the *SCN1A* variant was inherited from a healthy father. The proband had resistance to multiple antiseizure medications but responded well to sodium channel inhibitor Carbamazepine. Reanalysis of NGS data by a neurogeneticist revealed a previously uncharacterized heterozygous variant c.1035–7A>G in the *SCN2A* gene. Minigene assay showed that the c.1035–7A>G variant activates a cryptic intronic acceptor site which leads to 6-nucleotide extension of exon 9 (NP_066287.2:p.(Gly345_Gln346insTyrSer). *SCN2A* encephalopathy is a recognizable severe phenotype. Its electro-clinical and treatment response features can serve as a hallmark. In such a patient, reanalysis of genetic data is strongly recommended in case of negative or conflicting results of DNA analysis.

## Introduction

Febrile seizures (FS) occur in about 2–3% of children from 3 months to 5 years of age with a phenotype outcome that varies from simple febrile seizures to Dravet-like syndrome and epileptic encephalopathy (Sharkov, 2020). Atypical features of febrile seizures such as focal semiology, prolonged episodes (>15 min), or multiple seizure types within the same fever episode are of increased risk of developing epilepsy. After the third febrile seizure, the risk of additional episodes is approaching 50% and the risk of epilepsy formation is up to 15.8% (Bertelsen et al., 2016). Recent studies showed the contribution of genetic causes in the development of febrile-associated epilepsy including inborn errors of metabolism (mitochondrial, peroxisomal, lysosomal diseases, organic acidurias, aminoacidopathies, glycosylation, and urea cycle disorders), monogenic early-onset epileptic encephalopathies, and epilepsy phenotype caused by copy number variations (CNVs) such as the Cornelia de Lange, Seckel, and Rubinstein–Taybi syndromes (Dadali et al., 2016; Wang et al., 2017; Deng et al., 2018).

Febrile onset of epilepsy or seizure exacerbation during febrile/afebrile illness is observed among patients with pathogenic variants in genes that are associated with epileptic and developmental encephalopathies (DEEs) including *PRRT2*, *STX1B*, and *PCDH19*, and genes encoding voltage-gated sodium channels such as *SCN1A*, *SCN2A*, *SCN3A*, and *SCN8A* are known as the cause for fever-associated seizures or epilepsy (Deng et al., 2018; Yokoi et al., 2018; Shibata et al., 2021).

However, the *SCN1A* gene is the most common cause of febrile-associated epilepsy among early-onset DEE (Verbeek et al., 2013; Symonds et al., 2019). *SCN1A*-related epilepsy is often represented by Dravet syndrome (DS) which typically presents in the first year of life in a normal child with prolonged, afebrile and febrile (triggered by mild fever or hot bath), focal (usually hemiclonic), and generalized tonic–clonic seizures. The first seizure occurs before 12 months of age in over 90% of cases, usually between 4 and 8 months (Gataullina and Dulac, 2017). Yet in rare cases, a later onset of seizures was described in patients up to 18–20 months of age (Wirrell et al., 2017).

The onset of myoclonic seizures is typically by the age of two in most cases. Non-convulsive status, focal seizures with impaired awareness, and atypical absence seizures generally occur after 2 years. Between 1 and 5 years of age, in the “worsening stage” of DS, motor seizures become more frequent but shorter, although their severity is still linked to mild hyperthermia.

Patients with DS have normal development prior to seizure onset, and neurological abnormalities typically become evident in 3–4 years of age and include crouched gait, hypotonia, incoordination, and impaired dexterity. Seizures are typically exacerbated with the use of sodium channel blocking drugs such as carbamazepine, oxcarbazepine, phenytoin, and lamotrigine (Gataullina and Dulac, 2017; Wirrell et al., 2017).

Here, we report a patient with epileptic and developmental symptoms similar to those of *SCN1A*-related encephalopathy but with an unusually good response to sodium channel blockers and his diagnostic odyssey.

## Results

### Case Presentation

A five-year-old girl, the first child of unrelated healthy parents, developed focal epilepsy at 18 months of age following a normal pregnancy and delivery. There was no family history of any neurodevelopmental disorders or epilepsy.

Autistic features and mild speech delay were recognized after 12 months and included poor eye contact, stereotypic movements, poor communication skills, and delay in the formation of joint attention. She spoke two to three simple words at the age of two.

**FIGURE 1:**
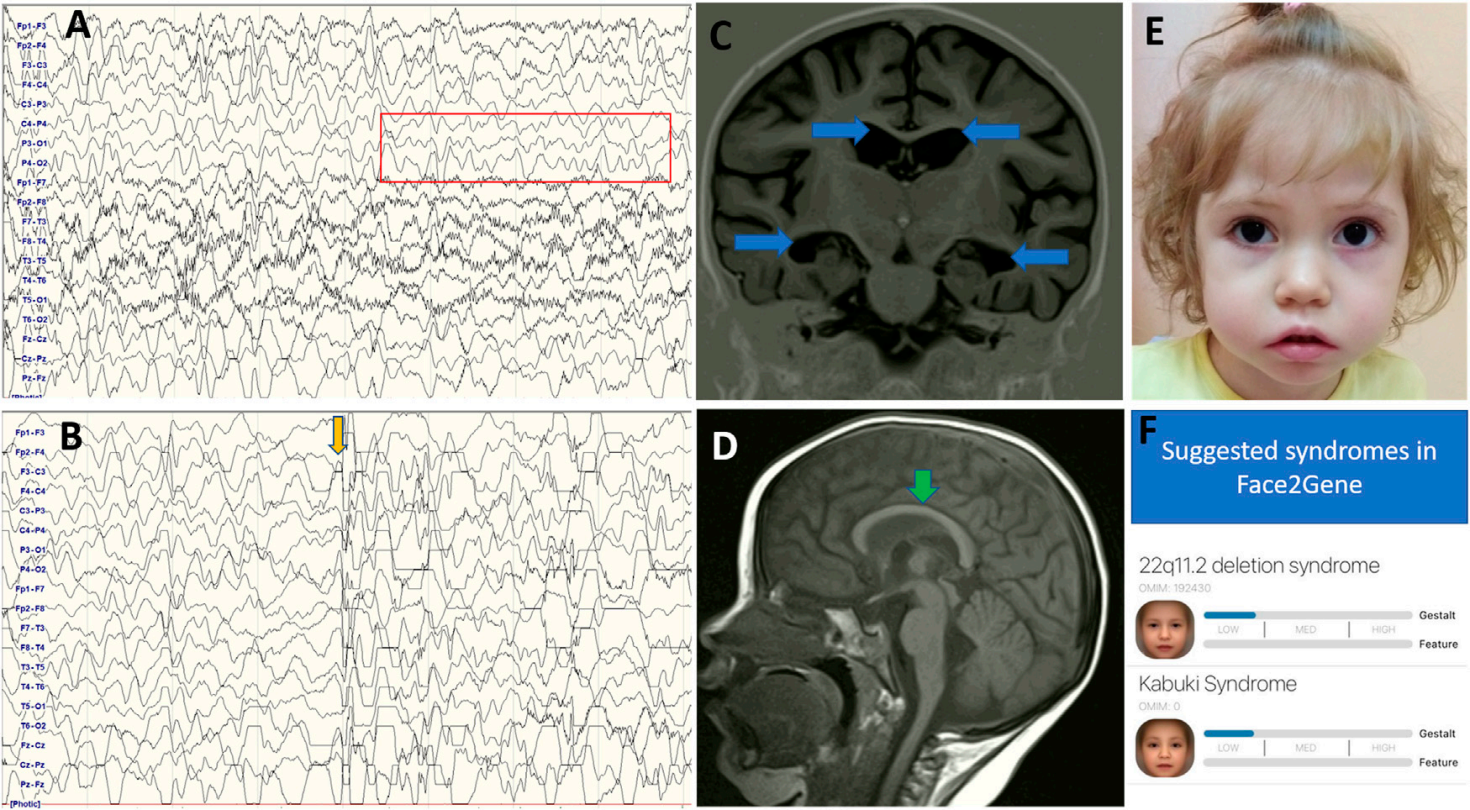
EEG longitudinal bipolar montage. Sweep: 30 mm/s; sensitivity: 150 mV/mm; bandpass: 1–70 Hz. Awake EEG **(A)** showing slow background activity with normal posterior dominant rhythm. Ictal EEG recording **(B)** showing myoclonic jerks with diffuse discharge and bursts of slow waves. 1.5T axial **(C)** and sagittal **(D)** T1 MRI indicated a mild diffuse brain atrophy, bilateral ventriculomegaly (blue arrows), and thinning of the corpus callosum (green arrows). Facial features **(E)** including a pronounced double curve of the upper lip (Cupid’s bow) and long palpebral fissures. Face2Gene analysis **(F)** showing low overlap with Kabuki syndrome.

Her psychomotor development had been considered normal until epilepsy onset which was characterized by bilateral tonic-clonic seizures, lasting 1 min, recurring several times within 2 days, and then increased in frequency to five seizures per day. An electroencephalogram (EEG) showed regional interictal spikes in the right central-frontal area (Figure 1A).

Several antiseizure medications (ASMs) were used in different combinations:

• Levetiracetam (LEV) and carbamazepine (CBZ) stopped seizures for 6 months and improved the cognitive skills of the patient. After LEV was removed rare seizures appeared again.
• Topiramate (TPM)—led to cognition deterioration.
• Phenobarbital (PB)—led to severe weakness, drowsiness.
• CBZ with LEV and lamotrigine (LMT) decreased seizure frequency.
• Ethosuximide (ETX)—had no effect.
• Сlonazepam (CLZ)—had no effect and proved to be poorly tolerated.
• Valproate (VPA)—aggravated myoclonic seizures.
• Hormonal treatment (ACTH)—aggravated the seizures and led to agitation.

At a follow-up at the age of 2 years and 2 months, the proband displayed severe developmental delay and an increasing number of seizures up to multiple daily. She had almost a complete skills regression as she was not able to sit or walk independently and was non-verbal. The proband had both febrile provoked (triggered by mild fever) and daily afebrile generalized tonic, tonic-clonic, myoclonic, and myoclonic-atonic seizures, with rare status epilepticus. At the last clinical examination, the proband was 5 years old and had autistic features, suffered from severe cognitive impairment, and was unable to speak. She has mild dysmorphic features such as long palpebral fissures and tented upper lip but without eyelid ectropion, prominent fingertip pads, or major visceral anomalies or dysfunction.

Neurological examination showed muscle hypotonia with high tendon reflexes, discoordination, and ataxia. She had focal tonic (versive) seizures that persisted with secondary generalization that affected both sides alternately, with a frequency of one to two times a week. Fever occasionally aggravated seizure frequency up to one episode a day. There was no association of the seizures with sleep.

Slow background activity with normal posterior dominant rhythm, bifrontal and generalized discharges (sometimes followed by electrodecrement), low index during awakeness, and low-average index in the sleep appeared on EEG at 4 years of age (Figure 1B). Ictal EEG recorded short generalized tonic seizures and myoclonic jerks with diffuse discharge and burst of slow waves.

Brain MRI indicated mild diffuse brain atrophy, bilateral ventriculomegaly, and thinning of the corpus callosum. (Figures 1C,D).

### Genetic Analysis

Due to the bilateral febrile provoked nature of the seizures, a monogenic etiology was suspected and an NGS-based custom gene panel of 2088 genes associated with epilepsy was performed. An in-house software pipeline was used designed to detect single-nucleotide variants (SNVs). The initial analysis revealed a heterozygous undescribed frameshift variant in the *KDM6A* gene—NM_021140.4:c.2831dupA (p.(Tyr944*)). The variant was classified as pathogenic according to the ACMG guidelines (Richards et al., 2015). Loss of function (LoF) variants in the *KDM6A* gene are associated with Kabuki syndrome (KS) 2 where haploinsufficiency is the main molecular mechanism (Lederer et al., 2012). The second reported variant was a heterozygous previously described missense variant in the *SCN1A* gene—NM_001165963.4:c.379C>G (p.(His127Asp)) (Zuberi et al., 2011; Cetica et al., 2017; Lindy et al., 2018; Brunklaus et al., 2020; Minardi et al., 2020). The variant affected a conserved amino acid residue where two other missense variants were reported as pathogenic in the literature (Xu et al., 2015; Djemie et al., 2016). The c.379C>G variant was predicted to be deleterious by several bioinformatic predictors including MutationTaster, PrimateAI, SIFT, and BadMut (Sim et al., 2012; Schwarz et al., 2014; Korvigo et al., 2018; Sundaram et al., 2018). According to the gnomAD database, the variant was observed 20 times in a heterozygous state with an allele frequency of 0.00007092. According to the ACMG guidelines, the variant was classified as a variant of uncertain significance (VUS).

Segregation analysis confirmed the *de novo* status for the variant in the *KDM6A* gene while the variant in the *SCN1A* gene was inherited from a healthy father with no clinical history of epilepsy (Supplementary Figure S1).

### X-Chromosome Inactivation

Due to the fact that KS type 2 is an X-linked dominant syndrome, several studies linked the varying expressivity in females with skewed X-chromosome inactivation (XCI) (Lederer et al., 2014; Kim and Lee, 2017). To explore the possibility of a skewed XCI pattern in our patient we performed methyl sensitive quantitative fluorescent PCR (QF-PCR) of the polymorphic repeat (CAG)n in exon one of the *AR* gene. We observed almost completely skewed inactivation in the patient with an XCI ratio of 98:2 (Supplementary Figure S2).

### Reanalysis of NGS Data

Reanalysis of the NGS data by neurogeneticists revealed a novel heterozygous intronic variant in the *SCN2A* gene - NG_008143.1(NM_021007.3):c.1035–7A>G which was classified as VUS. The variant was not found in the gnomAD database and it was predicted to not affect splicing by DANN (Quang et al., 2015). The nucleotide position appeared to be not strongly conserved. Moreover, another nucleotide variant located in the same position (c.1035–7A>C) was found 42 times in a heterozygous state in gnomAD with an allele frequency of 0.0001671. On the other hand, other splicing predictors suggested a potential impact on splicing including ADA (0.99924302), HSF 3.1 (+61,22%), and SpliceAI (acceptor gain: Δ score 0.91) (Desmet et al., 2009; Jaganathan et al., 2019). Sanger sequencing was performed in the family and confirmed the *de novo* status of the c.1035–7A>G variant in the *SCN2A* gene (Supplementary Figure S1).

### Minigene Splicing Assay

**FIGURE 2:**
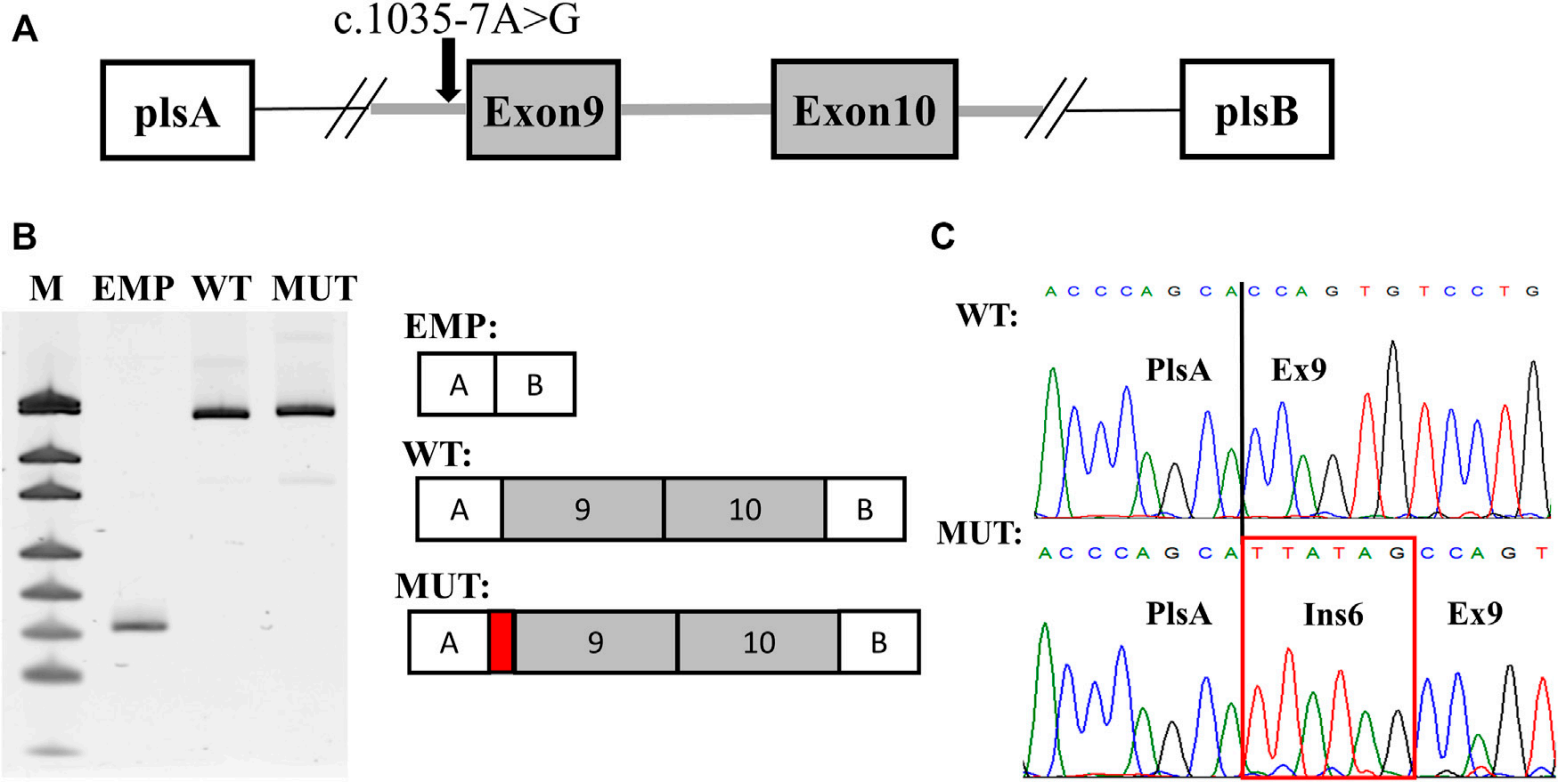
Results of the splicing minigene assay. **(A)** scheme of the minigene plasmid. Red arrow indicates the c.1035–7A > G variant. **(B)** plasmid-specific RT-PCR products in PAGE with urea. WT—wild-type isoform. MUT—mutated isoform. EMP—PCR product from an empty plasmid used as a control. M—pUC19 DNA molecular weight ladder. **(C)** Sanger sequencing of the detected isoforms. The 6-nucleotide extension of intron eight is highlighted by a red rectangle.

For functional characterization of the c.1035–7A>G variant and due to the fact that *SCN2A* gene expression is brain restricted, a splicing minigene assay was performed. Exon 9, intron 9, and exon 10 with the adjusted intronic regions of the *SCN2A* gene were amplified from the proband genomic DNA and cloned into the pSpl3-Flu2 splicing vector (Sparber et al., 2020). Wild-type (WT) plasmid and a plasmid carrying c.1035–7A>G variant (MUT) were separately transfected to HEK293T cells and 48 h post-transfection total RNA was extracted and RT-PCR was performed. RT-PCR using plasmid-specific primers showed a single product in both WT and MUT constructions, however, with a slightly larger MUT fragment seen in PAGE (Figure 2B). Sanger sequencing revealed normal splicing pattern in the WT construction with the inclusion of both exons 9 and 10. In the MUT construction splicing alteration was noted. The c.1035–7A>G variant activated a cryptic intronic acceptor site with elongation of intron 8 by 6-nucleotides—p.(Gly345_Gln346insTyrSer) (Figure 2C). This insertion of two amino acids affects the extracellular domain of NaV1.2 between S5 and S6 in repeat one where several pathogenic missense variants were previously described in patients with *SCN2A*-related epilepsy (Shi et al., 2009; Wang et al., 2012; Wolff et al., 2017; Fernandez-Marmiesse et al., 2019). Based on the results of the minigene assay the c.1035–7A>G was classified as likely pathogenic (PM2, PM4, PP3, and PS2).

## Discussion

Patients with KS have a pathogenic or likely pathogenic variant in *KMT2D* or *KDM6A* genes and distinctive facial features. The most commonly occurring features of KS are long palpebral fissures, eyelid ectropion, and fingertip pads. A large majority of people with KS are mildly or moderately intellectually disabled from birth, although some patients may have severe intellectual disability (ID). In a minority of patients the intelligence quotient (IQ) may be within the normal range as reported in (Matsumoto and Niikawa, 2003).

Boniel et al. demonstrated that patients with KS generally have low IQ levels and upon testing with the CARS, ADOS, and ADI-R scales (used in autism spectrum disorders), they tend to fulfill the criteria for autism. The prevalence of epilepsy in patients with KS has varied between 5 and 16–36% according to different sources. Seizure types most commonly include focal seizures, bilateral tonic–clonic seizures, and myoclonus. There are no reports of patients with KS and epileptic encephalopathies (Boniel et al., 2021).

Although, the segregation analysis confirmed the *de novo* status of the c.2831dupA variant in the *KDM6A* gene and the variant is predicted to lead to LoF which is in agreement with the molecular mechanism of KS we believe that the diagnosis could not be confirmed based solely on genetic data. The absence of typical clinical signs of KS and signs of epileptic encephalopathy in the proband raise doubts concerning its causality. The patient had only mild dysmorphic facial features (Figure 1E), had normal motor and near-normal developmental milestones until seizures onset, and had none of the supportive symptoms according to the international consensus diagnostic criteria of KS (Adam et al., 2019).

This incomplete penetrance in our patient could be explained by the almost complete skewed XCI, which is in agreement with previously reported clinically unaffected female carriers of a pathogenic variant in *KDM6A* (Lederer et al., 2014; Khodaeian et al., 2021). Several studies reported that even though the *KDM6A* gene escapes inactivation the expression level from the inactivated X-chromosome is significantly lower (Xu et al., 2008; Lederer et al., 2012). In this scenario, in our patient, there are near-normal levels of WT *KDM6A* protein which can rescue the phenotype. However, we could not fully exclude that the frame shifting variant in *KDM6A*, even with such skewed XCI may affect and modify the epilepsy phenotype in our patient.

The second reported variant p. His127Asp in the *SCN1A* gene was previously described in patients with DS and generalized epilepsy with the febrile seizures plus (GEFS+) phenotype (Zuberi et al., 2011; Cetica et al., 2017; Lindy et al., 2018; Brunklaus et al., 2020; Minardi et al., 2020). Nowadays, DS can be diagnosed among patients who met at least four of the five inclusion criteria: 1) normal or near-normal cognitive and motor development before seizure onset; 2) ≥2 febrile or afebrile seizures before 1 year of age; 3) seizure semiology consisting of myoclonic, hemiclonic, or generalized tonic-clonic seizures; 4) ≥2 seizures lasting longer than 10 min; and 5) failure to respond to first-line antiepileptic drug therapy with continued seizures after 2 years of age (Wu et al., 2015).

An additional feature of *SCN1A*-DS is an exacerbation of seizures during the use of sodium channel blocking drugs. Our patient had an unusually good response to carbamazepine. Also, we did not observe a non-convulsive status and atypical absence which generally occur after 2 years. Moreover, the proband had severe muscle hypotonia with increased tendon reflexes which is also rarely observed in *SCN1A*-related epilepsy.

Based on the fact that the variant in the *SCN1A* gene was observed 20 times in a heterozygous state in the gnomAD database, the fact that the proband father was an unaffected carrier and that the clinical picture was unusual for *SCN1A*-related epilepsy a different genetic etiology was suspected.

The favorable response to sodium channel inhibitors was suggestive for the *SCN2A* gene where in the previous analysis the intronic variant was missed. *SCN2A* encodes the pore-forming protein type 2 alpha subunit Nav1.2 of neuronal voltage-gated sodium channels. NaV1.2 is widely expressed throughout the human central nervous system, predominately in excitatory, glutamatergic neurons. It is located in the initial segment of the axons and is involved in the initiation and propagation of action potentials in a range of neuron classes (Boiko et al., 2003; Sanders et al., 2018).

*SCN2A* pathogenic variants have been identified as a prominent cause of a wide range of conditions, from benign neonatal or infantile seizures to neurodevelopmental disorders, including ASD, ID, and infantile-onset seizures of varying severity (Syrbe et al., 2016).

Also, few patients with unusual courses of diseases such as schizophrenia (Carroll et al., 2016) and recurrent ataxia (Leach et al., 2016) were reported.

*SCN2A*-epilepsy was divided into two groups depending on seizure onset: early infantile epilepsies (<3 months) and later onset (≥3 months), and described favorable effects to sodium channel blockers. Even in the late-onset epilepsy group, such medications were less effective and in some cases, induced seizure worsening (Wolff et al., 2017). Some patients with *SCN2A*-epilepsy have seizure exacerbation with intercurrent febrile or afebrile illnesses, including Dravet-like syndrome phenotype (Shi et al., 2009).

Reanalysis of the NGS data by neurogeneticists revealed a novel heterozygous intronic variant in the *SCN2A* gene: c.1035–7A>G. Following analysis of the variant has shown his potentially causative role and explained the course of the disease. Overall, based on the atypical clinical picture, lack of cosegregation with the previously reported variant in the *SCN1A,* and the result of the functional analysis which are concordant with the molecular pathogenesis of *SCN2A*-related DEE we believe that the intronic variant is causative in the reported patient.

Reanalysis of NGS data is an important tool in cases with no causative variants (Liu et al., 2019). An additional investigation by a novel specialist could dramatically increase the diagnostic yield, not only by finding causative variants in novel, previously undescribed genes, but also by finding variants that were filtered out during the first interpretation. In such cases, deep phenotyping and detailed clinical examination are of great importance.

A major challenge in DEE is establishing the pathogenicity of novel nucleotide variants and performing robust genotype-phenotype correlations. Considering the genetic heterogeneity of DEE, functional analysis is an important tool that helps in understanding the molecular consequences of the genetic variant and is crucial for proper genetic counseling. Unlike low-throughput, time-consuming approaches for investigation of the molecular defect on the protein level, for some variants, functional analysis on the RNA level can be used as an effective alternative (Wai et al., 2020). In such cases, the major limitation is the expression level of the gene of interest in an available biological sample. However, even for genes with tissue-specific expression patterns such as *SCN2A* for a subset of variants that are predicted to affect splicing functional analysis using the minigene assay can overcome this limitation.

Splicing variants are more and more recognized as a major cause of Mendelian disorders. Some estimate that up to 50–60% of all pathogenic variants in monogenic diseases could in fact be splicing variants (Wai et al., 2020). Abnormal splicing is more often associated with LoF, due to frameshift and nonsense-mediated decay activation. Yet, the exact molecular mechanism of splicing variants is very challenging to predict, given the complexity of the splicing machinery. In such cases, functional analysis using different approaches such as the minigene assay is crucial for a proper understanding of the molecular pathogenesis. In our patient, the splicing change leads to the insertion of two amino acids and does not alter the reading frame. This observation fits with the molecular mechanism of *SCN2A*-related DEE, where LoF variants are often found in patients with intellectual disability and GoF variants are linked to epileptic phenotype (Wolff et al., 2019).

Here, we report a diagnostic odyssey of a patient with an atypical Dravet-like phenotype, but with a good response to sodium channel inhibitors. Reanalysis of genetic data and functional analysis confirmed the causative role of the undescribed intronic variant in the *SCN2A* gene c.1035–7A>G. This report highlights the importance of a phenotype-driven strategy in DEE diagnosis and broadens the mutational spectrum of *SCN2A* epileptic disorders.

## Materials and Methods

Subjects: The proband was clinically examined in the Veltischev Research and Clinical Institute for Pediatrics of the Pirogov Russian National Research Medical University, Russia. Genetic analysis was performed in Genomed Ltd., Russia. Functional analysis was performed in the Research Centre for Medical Genetics., Russia. We reviewed medical files, EEG tracing, video EEG recordings, magnetic resonance imaging (MRI), and seizure course during the follow-up ranging from 2 to 3.5 years.

### DNA Analysis

Through the manuscript RefSeq accession numbers NG_016260.1and NM_021140.4 were used for the *KDM6A* gene, NM_001165963.4 were used for the *SCN1A* gene, and NG_008143.1 and NM_021007.3 for *SCN2A*.

An NGS-based custom gene panel of 2088 genes associated with epilepsy using genomic DNA was performed on Illumina NextSeq 500 instrument in 2 × 151 bp paired-end mode to an average depth of minimum 98.7x. The libraries were prepared and enriched using Illumina Nextera Rapid Capture Exome Kit v1.2. All candidate variants were validated using Sanger sequencing.

### X-Inactivation Analysis at the HUMARA Locus

The unequal X-chromosome inactivation pattern was analyzed according to Allen et al. (1992). For this purpose, the X-linked HUMARA polymorphic repeat (CAG) n in *AR* gene exon 1 methylation pattern was detected using methyl sensitive quantitative fluorescent PCR (QF-PCR) with subsequent fragmentary analysis on the ABI3130xl Genetic Analyzer (“Applied Biosystems”, United States). The inactivated X-chromosome-carrying (with or without the mutant allele) cell percentage (XCI ratio) was evaluated according to the formula proposed by Bolduc et al. (2008).

### Minigene Splicing Assay

Exon 9, intron 9, and exon 10 with the adjusted intronic regions of the *SCN2A* gene were amplified from the proband genomic DNA. The PCR product was cloned into a pSpl3-Flu2 plasmid vector as previously described (Sparber et al., 2020). Sanger sequencing was used for the selection of clones carrying wild-type (WT) and NG_008143.1 (NM_021007.2):c.1035–7A>G variant (MUT). WT and MUT plasmids were transfected into HEK293T cells using Metafectene (Biontex) according to the manufacturer’s instructions. Total RNA was isolated from the cells 48 h after transfection using the standard Trizol-based method, treated with DNAse I (Thermo Scientific), and reverse transcribed using ImProm-II™ Reverse Transcription System (Promega). Plasmid-specific primers were used in PCR for the detection of possible splicing alteration. PCR products were analyzed by denaturing PAGE with 8M urea with further Sanger sequencing.

## Data Availability

The datasets presented in this study can be found in online repositories. The names of the repository/repositories and accession number(s) can be found at: https://www.ncbi.nlm.nih.gov/, SUB11135283, and SUB11135320.
