# Supplementary material for: Case Report: Phenotype-Driven Diagnosis of Atypical Dravet-Like Syndrome Caused by a Novel Splicing Variant in the SCN2A Gene
Source: Front Genet. 2022 May 31;13:888481. doi: 10.3389/fgene.2022.888481 (PMC9194094; doi:10.3389/fgene.2022.888481)
Supplement: Supplementary file 1 [file DataSheet1.DOCX]

**Supplementary data**


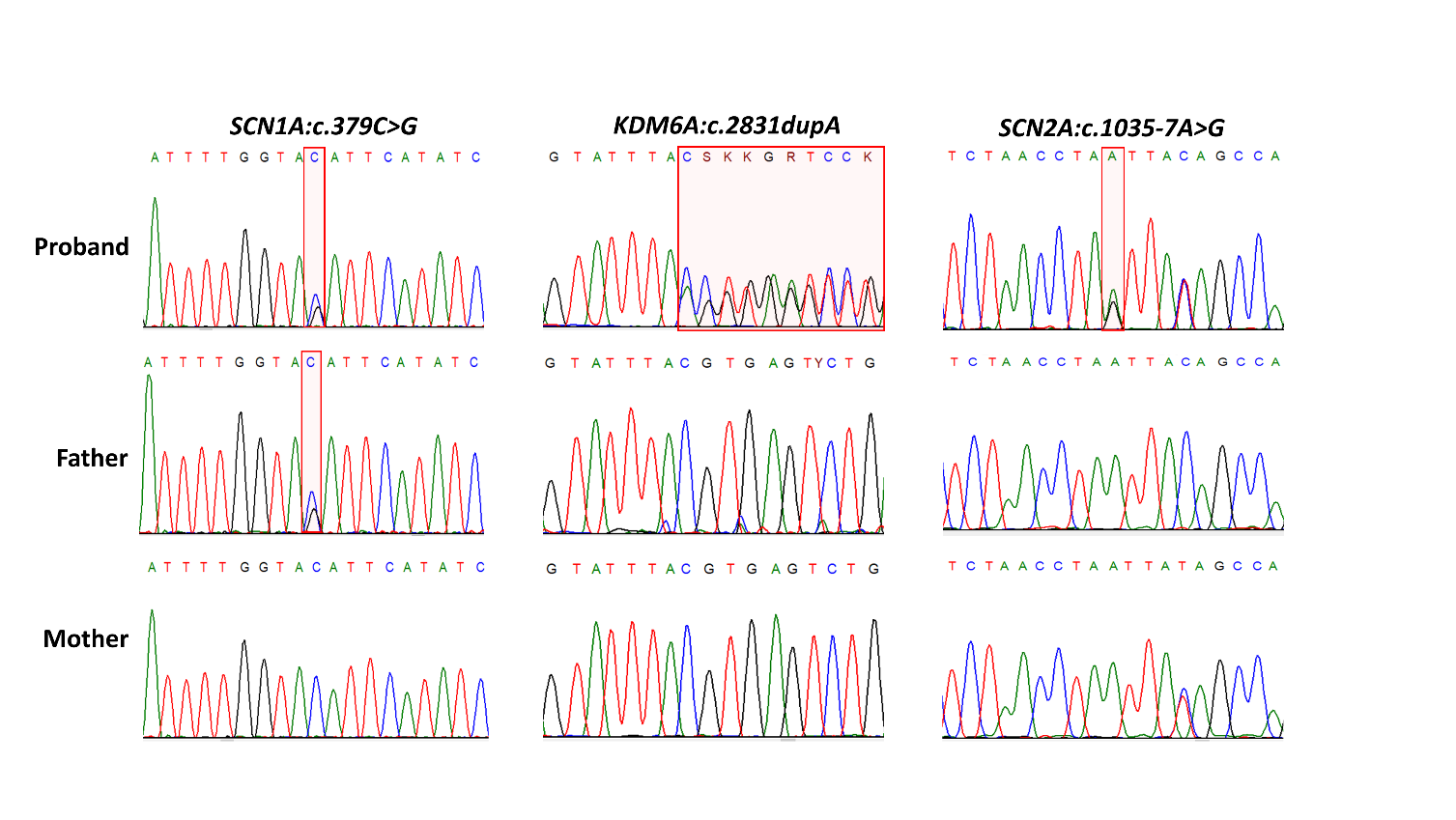


Supplementary figure 1. Segregation analysis of the c.379C>G in the *SCN1A* gene (left panel); the c.2831dupA variant in the *KDM6A* gene (middle panel); the c.1035-7A>G variant in the *SCN2A* gene (right panel). The variants are highlighted by a red rectangle.

**
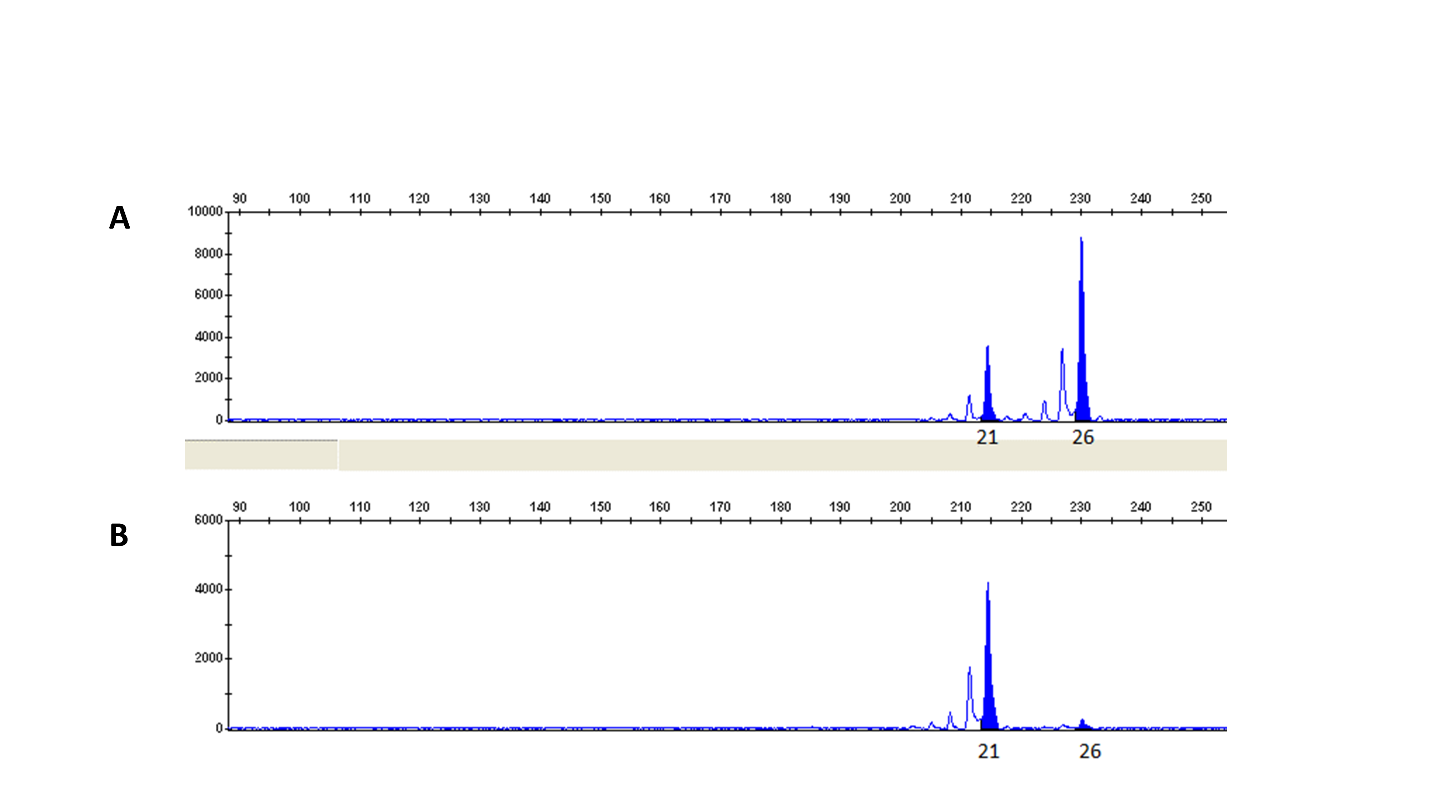
**

Supplementary figure 2. X-Inactivation Analysis at the HUMARA locus. Fragment analysis of the HUMARA polymorphic repeat ((CAG)n in *AR* gene prior to treatment with methylsensitive restrictase (A) and after treatment (B) showing near complete reduction of the 26 repeats peak.
